# Why has Not There been More Research of Concern?

**DOI:** 10.3389/fpubh.2014.00074

**Published:** 2014-07-22

**Authors:** Brian Rappert

**Affiliations:** ^1^Department of Sociology, Philosophy, and Anthropology, University of Exeter, Exeter, UK

**Keywords:** rationality, risk–benefit assessment, dual-use research of concern, precaution, biological weapons convention

## Abstract

Amid the renewed concern in the last several years about the potential for life science research to facilitate the spread of disease, a central plank of the policy response has been to enact processes for assessing the risks and benefits of “research of concern.” The recent controversy regarding a proposed redaction of work on the modification of a H5N1 avian influenza virus is perhaps the most prominent such instance. And yet, a noteworthy feature of this case is its exceptionalness. In the last 10 years, life science publishers, funders, and labs have rarely identified any research as “of concern,” let alone proposed censors. This article takes this experience with risk assessment as an invitation for reflection. Reasons for the low number of instances of concern are related to how the biosecurity dimensions of the life sciences are identified, how they are described, how the assessments of benefits and risks are undertaken, how value considerations do and do not enter into assessments, as well as the lack of information on the outcomes of reviews. This argument builds on such considerations to examine the limitations and implications of the risk–benefit experiment of concern framing, the politics of expertise as well as the prospects for alternative responses.

## Introduction

Throughout recorded history, attempts have been made by some to stop others from acquiring means of inflicting harm. From sixth century BC, efforts to check the spread of the formula for Greek fire to twentieth-century efforts to restrict designs for atomic and nuclear weapons, groups, and nations have exerted themselves to limit the potential for the diffusion of destructive capabilities – sometimes with specific users in mind, sometimes simply to anyone else. Attempts at control have extended far beyond weaponry itself. In different ways, natural resources, animals, information, and individuals have been subject to restriction, sanction, and suppression. Such attempts have been conceived in response to the hopes, events, fears, and preoccupations of their times.

Particularly since 9/11 and the subsequent anthrax postal attacks in the US, research in the life sciences has become an object of apprehension in relation to who might use it for what purposes. The question of how to prevent the life sciences from becoming the death sciences has been posed and answered in ways that raise questions for longstanding preoccupations and practices. Attention has extended beyond the access to pathogenic agents to also include scrutiny of what can be called “information products.” For instance, a central plank of recent biosecurity-related responses has been to develop processes for assessing the outputs of experiments. Much of this attention has been couched in terms of the imperative to weigh risks and benefits of openness. For instance, since 2003 a number of civilian science journals have established procedures for reviewing individual submissions in relation to whether “the potential harm of publication outweighs the potential societal benefits” (1).

This and similar activities undertaken by funders, university departments, and others have prompted wide-ranging discussion, typically framed in terms of where the balance should be struck between scientific freedom and national security. Much debate, sometimes heated, has taken place about the appropriateness of restricting what research gets done and how it is communicated.

Interestingly, though it is widely acknowledged that almost any knowledge and techniques in the life sciences can be used for destructive purposes, in practice it has been rare that risk assessments have identified anything as “of concern”; meaning that it poses clear possibilities for harm. It has been much rarer still that the harms of research have been deemed to outweigh its benefits.

This article takes this experience as an invitation to question how and why this is the case. The argument is divided into six sections. The next section recounts the recent history of attention to the security implications of the life sciences, with particular reference to the identification and assessment of “research of concern” and related designations. As will be argued, despite the limited identification of concerns and frequent expression that weighing the future benefits and risks associated with individual instances of research is not feasible, the enacting of assessment procedures remains a central strain of current international biosecurity efforts. The third section then asks how it is that the measures enacted to spot concerns rarely do so.

The fourth section elaborates on the pervasive but tension-ridden notions of “rationality” that underpin the assessment of experiments of concern. The fifth section offers alternative ways of conceiving of concerns associated with the destruction implications of the life sciences. These speak to issues about the politics of expertise. In particular, it will be argued that rethinking the terms of the present debate enables new possibilities for understanding the relation between science and society as well as the place of precaution in biosecurity.

## A Recent History of Concern

Regard for the link between the production of knowledge and the capabilities for inflicting disease has a long history. A recurring theme of much of the previous century and a half of modern biology has been the manner in which the latest understanding of disease fed into state and other biological weapons programs (2). This section elaborates how such regard has led to the recent notion that research might be “of concern.”

To begin with, it can be noted that proposals for controlling intangible knowledge and information did not figure prominently within Western life science policy discussions in past decades. For instance, in the years prior to 9/11, many analyses considered the new destructive possibilities enabled by developments in biology and related fields (3–5). Proposals for what needed to be done centered on strengthening physical controls on the transfer of pathogen agents and who has access to them. In this vein, in the immediate aftermath of 9/11 and the US anthrax letter attacks, initial legislative measures (such as the 2001 US *PATRIOT Act* and the later *Public Health Security and Bioterrorism Preparedness and Response Act of 2002*) enhanced requirements on the registration, movement, storage, and use of deemed dangerous bioagents as well as who could legitimately access them (6). Similar controls were introduced in a number of other countries.

Of note then, post-9/11, there have been suggestions that the outcomes of fundamental research might need to be scrutinized and restrictions imposed because of their security implications. As an example, in late 2001 the former head of research at SmithKline Beecham, George Poste, in the role of chair of a US Department of Defense task force on bioterrorism called on biology to “lose its innocence” regarding its security sensitivities (7). For him that meant enacting procedures for vetting, classifying, or otherwise restricting what research gets done and published. Similarly, at that time Epstein examined the possible contribution of civilian science for enabling destructive capabilities. He offered the category of “contentious research” to denote “fundamental biological or biomedical investigations that produce organisms or knowledge that could have immediate weapons implications, and therefore, raise questions concerning whether and how that research ought to be conducted and disseminated” (6).

A prime example of the type of research that raised questions for both Poste and Epstein was the early 2001 publication detailing how Australian scientists inserted the interleukin-4 gene (IL-4) into the mousepox virus as part of efforts to devise a contraceptive for rodent populations (8). This manipulation resulted in a modified mousepox with significant mortality rates for non-immunized, immunized, and genetically resistant mice. The worry was that the publication of these results could provide a technique for enhancing the lethality of other pox viruses, including smallpox. Like others at the time, both Poste and Epstein also voiced apprehension that if scientists did not initiate a discussion about what controls might be needed for security sensitive knowledge, then they risked others imposing draconian measures on them.

At least in the US, efforts were made during 2001–2003 to set in place a potential basis for restricting research findings because of how they might aid bioterrorism. The *Homeland Security Act of 2002* included the requirement that US government agencies “identify and safeguard homeland security information that is sensitive but unclassified” (9); a provision that was feared would be applied to basic science. One discussion about the potential for restricting publications identified likely problems and stipulated that any system of publication review should have the “support of the international scientific community, which must perceive that the security benefits of restricting open publication outweigh the possible costs to science” (10).

At the time, there was little evidence of such widespread support. As previously mentioned, in early 2003 an informal group of 32 largely American based journal editors agreed voluntary guidelines for reviewing, modifying, and if necessary rejecting research articles where “the potential harm of publication outweighs the potential societal benefits.” (1) Yet this enactment went hand in hand with expressions of apprehension – not least voiced by those signed up to the guidelines – that security motivated restrictions or oversight measures might unduly jeopardize the advancement of science (11–14). A common refrain expressed both by those with roles in national security agencies and in life science professional organizations was that security might well be compromised overall if the said free exchange of information underpinning research was hindered (15–18).

What would become arguably the most prominent statement about the potential for the techniques, methods, and knowledge generated through life science research to aid destructive purposes was given in late 2003 by a US National Academies report titled “*Biotechnology Research in an Age of Terrorism*” (19). It recommended extending existing (largely self-governance) mechanisms already in place in the life sciences. In relation to the themes of this article, one recommendation called for the initiation of a system of pre-project review for so-called “experiments of concern.” Seven such categories were specified in the report; this included research that would:

* demonstrate how to render a vaccine ineffective
* confer resistance to therapeutically useful antibiotics or antiviral agents
* enhance the virulence of a pathogen or render a non-pathogen virulent
* increase transmissibility of a pathogen
* alter the host range of a pathogen
* enable the evasion of diagnostic/detection modalities
* enable the weaponization of a biological agent or toxin

It was argued that work that fell in these categories should be reviewed by existing biosafety and recombinant DNA review procedures for its security implications. Echoing a theme prevalent elsewhere, the report recommended this while also noting the importance of not jeopardizing the norm of open communication in science.

Through the sorts of initiatives mentioned in the previous paragraphs emerged a sense of the potential security implications of the life science research outcomes and the need for oversight measures. Those notions largely emanated from the US and they were directed at discrete instances of research situated at the nexus of terrorism and biology. In other countries at the time, the “experiments of concern” framing would be varyingly taken up, rejected, or ignored (20).

In the years after 2001, just how much of a threat was really posed by research was subject to varying assessments informed by alternative criteria about what harms mattered as well as what lessons should be drawn from past history about the likelihood and severity of bioattacks (21). Despite such differences, calls for identifying and assessing sensitive knowledge at the time generally shared a number of features including: the stated need not endanger the benefits of science that are derived from its openness; the encouragement to scientists to act before controls was placed on them from elsewhere; and the object of scrutiny being the future risks and benefits associated with individual experiments.

In relation to the last point, regard was directed at a limited number of such instances. Besides the previously mentioned IL-4 mousepox research, other prominent experiments were the 2002 publications detailing the successful artificial chemical synthesis of poliovirus (22) and the comparison of a type of smallpox and its vaccine that suggested a means of increasing the vaccine’s lethality (23).

### Experience with assessments

The attempts to identify and assess sensitive knowledge noted above sought to establish key points at which to make determinations about whether specific instances of research should go ahead or be communicated; this based on their anticipated potential future harms and benefits. In the years that followed the initial articulations of the “experiments of concern,” this manner of framing the security implications of the life sciences would become more widespread within international policy discussion. For instance, after the publication of *Biotechnology Research in an Age of Terrorism*, a number of similar calls were made to put in place “harm–benefit” or “risk–benefit”-related reviews of research, such as the World Health Organization’s (WHO) *Life Science Research: Opportunities and Risks for Public Health* and the American Medical Association’s *Guidelines to Prevent Malevolent Use of Biomedical Research*. The British-based Biotechnology and Biological Sciences Research Council, Medical Research Council, and Wellcome Trust did adopt review procedures for grant applications that posed a potential for misuse in 2005 (24). Despite such developments, little public articulation was given to how such assessments could be or were being conducted in practice.

Following directly from one of the recommendations of *Biotechnology Research in an Age of Terrorism*, in early 2004 in the National Science Advisory Board for Biosecurity (NSABB) was formed to provide advice on oversight strategies, guidelines, and education regarding the handling of federally supported “dual-use” research. Included within its remit was the devising of criteria for identifying and evaluating the risks and benefits. In 2007 as part of the document *Proposed Framework for the Oversight of Dual-Use Life Sciences Research*, it offered a split between two kinds of science: “dual-use research” was used “to refer in general to legitimate life sciences research that has the potential to yield information that could be misused to threaten public health and safety and other aspects of national security such as agriculture, plants, animals, the environment, and material” (25). Since nearly all science could be used in this manner, NSABB offered another category of “dual-use research of concern” (DURC). This denoted “research that, based on current understanding, can be reasonably anticipated to provide knowledge, products, or technologies that could be directly misapplied to pose a threat to public health and safety, agricultural crops and other plants, animals, the environment, or material” (23).

Within the framework envisioned by NSABB, should Principal Investigators determine that they are conducting DURC research it would then be subjected to institutional risk review to assess: “the likelihood that the information might be misused; the potential impacts of misuse [and] [s]trategies for mitigating the risks that information from the research could be misused” (26) In this way, a general framework for the risk assessment of individual research instances was elaborated.

While the activities of NSABB and others in relation to the scrutiny of research results have generated public, policy, and ethical discussion about the dangers they pose for science (27–29), one notable feature of the reviews is how few publications, grant applications, or project proposals have been identified as posing concern. Take the time period following the initial articulations of the category of “experiment of concern.” In a sample of 16,000 manuscripts submitted to the journals of the American Society for Microbiology after they adopted the 2003 journal publication guidance, only 3 were subjected to additional biosecurity peer review. By the end of 2006, the Wellcome Trust reported having identified three proposals as requiring additional security scrutiny with none judged to pose an overall concern on balance (26). Also, a US National Research Council report titled *Seeking Security: Pathogens, Open Access, and Genome Databases* argued against the prospect of being able to identify genomic data with significant security worries (17). Even in the case of the 2005 publications related to the sequencing of the 1918 Spanish Flu virus (30) and its subsequent artificial reconstruction (31), the benefits were deemed to outweigh possible risks by the journals involved. It was such experience up until 2007 that lead NSABB to anticipate “few” cases would fit into the DURC category and therefore that the initial assessment of experiments by Principal Investigators should not be time consuming (32).

This overall pattern of finding little of concern has continued through until today (33). Between 2009 and early 2014, the Wellcome Trust has flagged only two applications to its funding committee for scrutiny in relation to their misuse potential, with both not being funded on the basis of their scientific merit rather than due to security concerns (David Carr, personal communication, 12 February 2014). Of the 74,000 biological submissions to the Nature Publishing Group between 2005 and 2008, only 28 were identified as having a dual-use potential, with none rejected for this reason (34). The Danish Centre for Biosecurity and Biopreparedness has licensed projects in the Denmark that produce new technologies of a directly weapons potential and has not identified any cases of DURC publications (John-Erik, personal communication, 29 January 2014).

Such an overall situation is remarkable within the context of the multi-billion dollar increase in biodefense research funding in the US after 2001, much of it supporting civilian research (35). This massive expansive directed funding toward the type of work that would likely be of concern, and yet few such instances have subsequently been identified in practice. The US National Institute of Allergy and Infectious Diseases of the National Institutes of Health (NIH) distributed much of the funding for biodefense research. Its director reportedly indicated that in recent decades, the NIH has never had an instance in which funded research was retroactively judged as having been funded or published improperly (36, 37). Instead of large number of diverse instances of research being flagged on a regular basis, since 2003 a limited list of several experiments have come to be repeatedly cited (38), the latest at the time of writing being the reverse genetics creation and then mutation of a virus resembling the 1918 Spanish Flu virus (39).

Such experience makes it important to note that while the *Proposed Framework for the Oversight of Dual-Use Life Sciences* and other initiatives outlined processes of assessment, they did not specify in practice how potential future benefits and harms could be assessed and weighed. At the time, perception of this gap led to calls for the development of new risk assessment tools, often couched in terms of the need for objective quantification of the likelihood and impacts of bioattacks (40, 41). Within the work of NSABB itself, belief in the prospect of rigorous and value neutral calculations have been made alongside recognitions that the evaluation of dual-use potential of research inevitably would be subjective (42).

While in practice, few experiments were being identified as posing significant security concerns until and after the launch of the 2007 *Proposed Framework for the Oversight of Dual-Use Life Sciences*, this has come alongside contentions that practicing scientists have been largely unaware of the malign applications of their research. The World Medical Association, the US National Academies, the British Royal Society, the International Committee of the Red Cross, the Wellcome Trust, the InterAcademy Panel, NSABB, the International Council for Science as well as others have argued that practitioners needed greater education about the potential dangers associated with their work (43). In theory at least, the need for such enhanced understanding left open the possibility that a different pattern of review outcomes might emerge once individuals possessed the requisite awareness.

Calls for greater education have not been restricted to scientists though. Another accompanying current of dual-use discussions has been the repeatedly expressed anxiety about public understanding. For instance, over the course of its deliberations the NSABB Communications Working Group expanded attention from the time of its creation on the security threats stemming *from* research to include the threats *to* research posed by public misconceptions (44).

### The exceptional case of H5N1

Between June 2007 and late 2011, NSABB’s *Proposed Framework for the Oversight of Dual-Use Life Sciences* faced an uncertain future waiting for an official response by successive US administrations. The attention to dual use transformed significantly in late 2011 when a set of experiments on the H5N1 influenza virus became high profile. At that time, two groups lead by Ron Fouchier at Erasmus Medical Center and Yoshihiro Kawaoka at the University of Wisconsin, Madison submitted manuscripts to *Science* and *Nature* respectfully related to the mammalian transmissibility of a strain of H5N1; specifically indicating how a genetically mutated form of the H5N1 influenza virus could become transmissibly airborne between ferrets (45, 46). Up until that time, H5N1 was only known to be transmittable through direct physical contact. Although exactly what had been demonstrated would become a matter of controversy, this work identified a possible casual link between genetic munitions and airborne transmission between mammals more generally.

National Science Advisory Board for Biosecurity reviewed the publications and concluded they should go ahead, but minus certain details so as to reduce their malign potential (47). In the wide-ranging debate that followed, a year long moratorium was initiated by a group of 40 flu researchers (48). Both these moves reignited debates about the security implications of the life sciences – typically framed in terms of whether the freedom of science should be jeopardized in the name of security. The WHO convened an international meeting in February 2012 that heard additional non-public information about the experiments (49). That meeting concluded that full versions of the articles should be published once issues associated with public messaging had been addressed. In response to the controversy, in March 2012 the US Department of Health and Human Services issued a revised policy for DURC life science research (50).

While this experience with H5N1 has come to dominant recent discussions associated with the governance of experiments of concern and spurred renewed attention to implementing review procedures (51–53), what is perhaps most notable is its *exceptionality*. It is exceptional both in relation to the recommendation to withhold details for security reasons and the extent of policy and public discussion that took place.

With regard to the former, the recommendation of restricting details was to be subsequently overturned. In late March 2012, NSABB was reconvened and reversed its decision in voting overall in favor of publishing revised forms of both disputed papers. In justifying this shift, the Board cited the availability of new information that reduced worries about the ability of the research to immediately enable malign capabilities and that increased its public health benefits (54).

The case of H5N1 is similar to other discussions about experiments of concern though in its fraught relation with risk–benefit assessment. In reversing its initial decision, for instance, NSABB contended that “The Board’s discussions were informed by the analytical frameworks that it previously developed for considering the risks and benefits associated with the communication of DURC.” (54) That framework was the 2007 *Proposed Framework for the Oversight of Dual-Use Life Sciences Research: Strategies for Minimizing the Potential Misuse of Research Information*. Yet, as previously mentioned, this framework did not specify how potential future benefits and harms could be assessed and weighed in practice. Instead, it laid out organizational processes for handling DURC instances.

As another strain of the troubled status of risk–benefit assessment, apprehensions about the way NSABB conducted the assessment of benefits and risks was given in a critical response letter to the NIH leaked to the press. With regard to one of the controversial papers (subject to a 12–6 split decision in favor of publishing at the March 2013 NSABB meeting), a Board member lamented:

I believe there was a bias toward finding a solution that was a lot less about a robust science- and policy-based risk–benefit analysis and more about how to get us out of this difficult situation. I also believe that this same approach in the future will mean all of us, including life science researchers, journal editors and government policy makers, will just continue to “kick the can down the road” without coming to grips with the very difficult task of managing DURC and the dissemination of potentially harmful information to those who might intentionally or unintentionally use that information in a way that risks public safety (55).

Some commentators would go further, drawing the conclusion that weighing benefits and risks in relation to DURC issues was not feasible (56). Yet elsewhere, belief continued to be placed on the need for “careful consideration of the scope and magnitude of the potential risks and benefits associated with the research proposal, evaluation of whether the risks outweigh the benefits, and strategies for mitigating potential risks” (57) – as stated in the early 2013, NIH guides for US Department of Health and Human Services’ framework for funding decisions on individual proposals involving highly pathogenic avian influenza H5N1 viruses.

International attention to devising processes for identifying and evaluating research along these lines continue. The need for DURC-type oversight frameworks has been made elsewhere, including by some governments as part of the Biological Weapons Convention (58).

## Why is There Nearly Nothing?

For more than a decade, attention has been cast to the potential destructive application of knowledge generated from life science research and what, if any, governance measures need to be in place to advert their realization. While varying in their specifics, the attention to what can generically be called “research of concern” indicates a movement beyond traditional biosecurity preoccupations about materials, equipment, and personnel.

The previous section though drew attention to some curiosities: despite the importance often attached to assessing concerns, in practice few such instances have been identified. Moreover, since 2003 it would appear that (in the end) in no case of civilian formal reviews have the risks been deemed to outweigh benefits. On the back of this track record, important questions can be asked, such as: “how is it that so little concern has been identified?,” “how is belief in the value of assessment processes maintained despite their apparent lack of implications?,” and “what alternative ways of understanding are possible?”

This section principally addresses the first of these questions. It does so by examining the identification and weighing benefits and harms in order to suggest why cases have not been identified.

### What are the objects of concern?

Consider first the basic framing given to what is of concern. Whatever their other differences, the varied attempts to establish research of concern have generally shared the bounding of evaluations around specific instances of research. Both within assessment procedures and educational material (59), this means attention gets cast at individual (or in some cases more than one closely related) research applications, experiment proposals, and submitted manuscripts. Such instances are envisioned as the holders of potentially sensitive knowledge.

With such a focus, signaling out one piece of knowledge as of concern requires being able to separate out its contribution to the general stock of knowledge from all others. As scientific and technical developments are typically cumulative accomplishes, this is often difficult. Against past attempts to contend that a particular set of findings raised concern, counter claims have been made that previous work was suggestive of or already indicated grounds for concerns (60–62). The less a distinctive break from what was previously known, the more difficult it becomes to justify any security apprehension.

In contrast, rarely in policy discussions to date have assessments been offered at lines or programs of work (63). Taking these as the object for scrutiny though arguably opens up a space for wider set of questions and possibilities. For instance, the publications in 2005 pertaining to the sequencing of the 1918 Spanish Flu virus and its artificial reconstruction were only the end culmination of a long line of funded and published research (64). As a result, it was possible to scrutinize the activities associated with the 2005 publications well before the results were sent to in *Science* and *Nature*. Instead of asking “should this particular experiment go ahead or be published?” alternative broader questions could include “what lines of research should be funded in the first place?” The latter is important to acknowledge because in situations of limited funding, choices are inevitably made about, which research to support and which to not (65). As such, when a WHO report on its 2013 DURC meeting stated:

Scientific research is conducted in virtually all countries and is critical to strengthening global response to all health threats and hazards, including those posed by naturally occurring and by accidentally or intentionally released biological agents. The only way to eliminate the potential for misuse of DURC is to not perform research. Such an extreme solution, however, is neither feasible nor advisable (66).

It arguably did not make a room for acknowledging that choices are routinely made to back some lines of research over others (65). For all the roads taken, there are many not pursued.

The limiting of attention to individual experiments or publications is also consequential for the identification of concerns because it generally directs attention toward the latest, and thereby often most technically sophisticated, expensive and thereby exclusive research. Because of this sophistication, doubts can be raised about how feasible that it is that other groups can reproduce the work (67). The resulting situation is one much more difficult to assess than if consideration were directed at what capabilities are becoming widely accessible.

### How are concerns identified?

Working within the common conceptualization of individual instances of research being the potential holders of concern, further questions can be asked of the assessment procedures and practices enacted to date.

As previously noted, a variety of organizations have underscored the importance of practicing scientists being cognizant of the destructive potential of their activities. Without this awareness, assessment procedures reliant on Principal Investigators to identify concerns could not function as envisioned. Against this need though, many empirical studies have indicated such an awareness is possessed by relatively few practitioners (68). Thus, the relative infrequency of the identification might be attributed to a lack of awareness. This consideration along with the conflict of interest associated with researchers judging their own work led the Center for International and Security Studies at Maryland to forward an oversight system that requires independent peer review to include those with scientific and security expertise (69).

The contingencies associated with how research is and is not identified as posing concern can be highlighted through examining the regard given to the potential of research both before and after periods of prominent attention. For instance, in the case of the early 2001 IL-4 mousepox publication, the Australian scientists involved have argued that work undertaken prior to 2001 by others and in follow-on work they performed *after* 2001 indicated how to enhance the lethality of viruses (70). Yet, professional and public regard for those developments has been muted.

Other grounds can be offered for suggesting formal reviews might be limited in how they determine concern. The comparison between formal reviews and informal practice is one such basis. In a 2007 survey undertaken by the US National Research Council and the American Association for the Advancement of Science (AAAS), AAAS members with an interest in the life sciences were asked about their familiarity and experiences associated with dual use. Nearly one in six indicated they had made some sort of change to their research – for instance, whether it was undertaken, with whom, and how it was communicated – because of worries that the knowledge, tools, or techniques might be used in bioterrorism. The low response rate (16% completed the survey in full) means the findings were not statistical representative. However, they signal a level of regard not being registered through the formal assessment procedures enacted by published, funders or organizations (71). The criteria individuals employ in making self-determinations about the potential of their work would be a likely important topic for understanding rates of identification.

A relatively prominent recent case of *researcher-initiated* restrictions was the publication in 2013 of a new type of botulinum neurotoxin designated as BoNT/H (72, 73). With no effective treatment for this form of botulism, the researchers decided to withhold the sequence data on BoNT/H from their write-up of the research until an antitoxin is developed. In this case, the authors first consulted with various US federal government agencies about the advisability of publishing these and then secured agreement from the journal to publish without the sequence data or their submission to the International Nucleotide Sequence Databases (74, 75).

### How are risks and benefits determined?

Even when concerns are recognized, determining the risks and benefits has proven highly taxing and would likely be so into the future.

One challenge is that assessments of risks and benefits vary considerable. For instance, based on lab observation research and interviews, Bezuidenhout has argued distinct ways of making sense of risks and benefits exist between scientists in sub-Sahara Africa and those prevalent in Western dual-use discussions to date (76). Within the former, dual-use risks were regarded as hypothetical, biosecurity harms were frequently defined in relation to gross lab deficiencies in local waste disposal, and the benefits of research were associated with its ability to address disease in the immediate term.

Another often identified challenge is the inability of the many of those associated with the life sciences to assess the potential for malign applications. In classic risk assessment models, the expected value of risk is taken as a function of the likely probability of an event times its consequences. In relation to formal reviews for research of concern, given how the objects of concern are typically defined, what is demanded then is a way of assessing the possibility that unspecified users would draw on individual sets of findings toward the development of an unfixed range of destructive capability in a time frame that is not specified. Then assessors need to determine the expected consequences of such an action against likely available countermeasures. A fully developed notion of threat would also require regard for the intent of potential users.

As many have contended, practicing scientists are often not knowledgeable about the capabilities or intent of those that might employ their work for hostile ends (6, 70). The same has been argued for those that typically make up biosafety committees in universities and elsewhere (56). In this regard, it should be underscored that what is required for assessing dual use is twofold: one, information about matters such as motivations and capabilities and two, a competency through methods, concepts, and theories to assess experiments (77).

The extent to which either dimensions can be grasped at all in the case of dual-use life science research appears an open question. Just how much information is available and could be made widely accessible about the motivations and capabilities of would-be users is unclear; especially given the relative dearth of bioattacks in recent years that might provide a (however tentative) baseline for future extrapolation (78, 79), the clandestine status of any existing state or sub-state bioweapon programs, and the focus in reviews given to cutting edge capabilities enabled by the latest science.

In addition, though, despite the aforementioned importance often attributed to devising methods for determining the security risks associated with research of concern, little by the way of detail have been given about how this could take place (69, 80). The absence of methods for determining risks is a particularly salient point in relation trying to make sense of concerns outside of traditional agents used within biowarfare programs.

As such, much of the consideration of research of concern could be characterized as taking place in conditions of “ignorance” – that is in conditions characterized by limitations in both information and methods for assessment (77).

Yet, a further sense of the difficulties of determining risks is evidenced in how security related implications should be interpreted. To start, as has been repeatedly argued in relation to the DURC designation developed in the US, “characterization of research as DURC should not be viewed pejoratively” (81), meaning it need not necessarily be stopped, censored, or otherwise restricted because it is determined to be “of concern.” But questions of interpretation go beyond this point of a non-negative evaluation. The identification of concern has heighted the *positive* value attached to research because of what it suggests for assessing threats and countermeasures (82, 83). A notable feature of many of the experiments of concerns of the last decade is how the initial work led to follow-on activities undertaken worldwide and justified on both scientific and biodefensive grounds. The identification of the need for such follow-on work has led some to express anxiety about the risks to society from *restricting* dual-use information (84).

Whereas the downside potential of research is widely regarded as difficult to assess and often subject to radically diverging evaluations, the contention that benefits can be expected to accrue (however, much in the future, however, indirectly) is a starting point for many commentaries (80). In short, research is categorically taken as “an essential public good” (85). While the certainty or even likelihood of research leading to health improvements has been queried elsewhere, such doubts are rarely voiced within dual-use discussions (86). The case of H5N1 was a notable exception in the manner in which detailed questions were raised about its utility (87).

### How are risks and benefits weighed?

In classical risk assessment models, once risks and benefits are identified, these should be weighed against each other so that a net assessment can be reached. In the case of research of concern, for instance, this is expressed in the manner some publishers have committed themselves to assessing whether “the potential harm of publication outweighs the potential societal benefits” (1). Given the “ignorance” that often characterizes determinations of dual-use risks though, undertaking such a weighing has and will likely be bedeviled by problems.

In theory at least, such a situation could lead to a range of possible outcomes. For instance, post-9/11 in the US [and elsewhere (88)], fears about low probability but high-consequence terrorist attacks justified a range of domestic anti-terrorism measures and military actions (77). Parallel uncertainties and unknowns in relation to research of concern could have resulted in sweeping restrictions. This, however, has not taken place.

What explains this difference between the types of responses made in previous years? One set of considerations would seem to be the basic presumptions informing weighing. For instance, as mentioned above the default position has been that risks with research of concern need to be substantiated, whereas the benefits from research are typically assumed (41). Another prominent set of presumptions is that life science research – in the absence of security related controls – is characterized by the free and open flow of information, that such a situation is vital for the scientific progress, and that therefore any attempt to move away from this default needs to be justified (80). A related corollary is that once knowledge has been generated, it is not possible to undo it or restrict its flow (89). With such widespread presumptions, controls are difficult to justify.

Both lines of thinking are arguably questionable though. Social studies of the practice of science have indicated how the exchange of information in research is frequently subject to negotiation and limitation in practice – not least because of commercialization goals (90). In addition, to subscribe to the view that knowledge once generated is simply “out” and uncontainable relies on a reduction of knowledge to abstract and explicit propositional statements. In contrast, it is possible to highlight the practical skills, understandings, and competencies necessary to reproduce and utilize specific research. These ways of knowing are crucial to many aspects of the production of biological and nuclear weapons and, as such, some scope exists to affect (and even reverse over time) the proliferation of capabilities (91).

As Buchanan and Kelley argue though, the very attempt to pitch risks and benefits against each other and ask how they can be “traded off” is consequential. Such an approach often discounts what does not fit under the heading of “open science” or “security.” As they argue, within the typical dual-use framing:

…it is the interests of only two parties that are likely to be strongly represented: scientists who fear constraints on the pursuit of knowledge, and government officials whose worst nightmare is a bioterrorist attack that could have been prevented. Therefore, one of the dangers of an overly simplistic framing of the ethics of biodefense is that it largely ignores or arbitrarily discounts values that have been central to the research ethics debate since its inception: the protection of research subjects, both human and non-human [i.e., animal] (92)

With this silencing, weighing is likely to be skewed.

This formulation of the limitations of dominant framings today itself though arguably makes questionable presumptions. As with much of the discussion about biosecurity generally and research of concern specifically, Buchanan and Kelley treat the issues at stake as subject to contention by two competing communities with distinct interests: those on the side of “science” and those on the side of “security” (93). It is the latter “security community” that is treated as seeking restrictions on what research gets done and how it is communicated. Appeals to such a community have been routinely evoked in dual-use discussions, though without defining its membership.

In practice, it is difficult to identify a coherent security community in relation to the specific topic of “research of concern,” let alone one that has worked in a concerted effort to imposing restrictions. This is the case both outside of the US (where, in general, dual-use concerns have been more muted and biosecurity expertise within national security communities is more limited) as well as in the US. Indeed, some of those raising the most significant worries about threats to science have been those that would likely be identified as part of “the security community” (89). In the absence of a coherent group consistently forwarding security-inspired restrictions, the track record of the last 10 years is not surprising.

### How has experience been evaluated?

In models for managing risk, much emphasis is often placed on scrutinizing experience and modifying assessments in response. As with the aforementioned components, here too points can be suggested about why there has been little research of concern.

One pertinent point is the lack of systematic data on how often experiments and publications of concern have been identified and the decisions reached as part of formal reviews. While some figures have been made available at meetings or in publications, and some analysts have complied information (33), the resulting picture of practice has been fragmentary and partial. Such a situation stifles learning from experience.

In this respect, an interesting feature of the discussion about this topic is how experience to date is often not taken as relevant to informing policy recommendations. For instance, in an otherwise wide-ranging and empirically rich analysis of the dual-use policies of biomedical journals, Resnik and colleagues lamented on the low rate of journals with such policies in place (94). To correct for this, they called for journals to develop such policies. Yet, this analysis did not seek to determine the implications (if any) of the reviews undertaken and thereby their practical relevance (95). Instead, the utility of reviews was assumed. In general, a lack of evidence about the results of reviews undertaken characterizes other prominent statements on this topic (63).

At least in relation to US federally funded research, the absence of information may change. In March 2012, the Federal government issued a policy titled “United States Government Policy for Oversight of Life Sciences Dual-Use Research of Concern.” It calls for a “regular review of United States Government funded or conducted research with certain high-consequence pathogens and toxins for its potential to be DURC in order to: (a) mitigate risks where appropriate; and (b) collect information needed to inform the development of an updated policy, as needed, for the oversight of DURC.” (96). Figures compiled by the NIH in early 2012 indicated 381 extramural and 404 intramural projects using high-consequence pathogens or toxins. Ten of the extramural projects and none of the intramural projects were designated as DURC (97). At the time of writing, however, it is unclear what information agencies in the US will release on the outcomes of reviews.

## Assessment and Rationality

Taken together, the previous sections suggested recent discussions about research of concern have been tension-ridden. On the one hand, much of the attention to this topic has been initiated in response to individual experiments, yet that object of scrutiny also delimits the scope for consideration. While a handful of instances of contentious research have served as prompts for wide-ranging calls to rethink the oversight of the life sciences, few other such examples have been identified and it has been exceedingly rare that risks have been deemed to outweigh benefits. Vocal, resolute, and repeated apprehension has been expressed about how security-initiated reviews threaten the scientific enterprise, and yet to date formal reviews have had seemingly little bearing on what activity gets done or how it is communicated.

Despite the divergent ways of making sense of whether and what kind of concern should be associated with the informational products of research, much of the discussion shares a common object for scrutiny and a common language for thinking about assessing concern: namely, a focus on weighing the future benefits and risks of individual elements of research. An often recurring assertion has been that the extent of concern can be rendered known, and thereby manageable, through rationalistic “risk–benefit” assessment procedures.

At times, highly ambitious goals have been ascribed to assessments. A 2009 Royal Society workshop report titled *New Approaches to Biological Risk Assessment*, for instance, suggested dual-use risk assessments need “to link epidemiological modeling of disease, economic modeling, and qualitative social science modeling of human behavior” (98). Moreover, it added, “public perceptions and media reactions play an important role in driving policymakers” decisions on biological risks, particularly in the context of risk management and communication. Therefore, any risk assessment methodology needs to encompass assessment of human behavior and motivations, and any model needs to incorporate feedback loops to address the public’s reaction to government risk management policies” (98). Achieving such aspirations for comprehensive rigor was said to require national and international harmonization through multidisciplinary analysis, a point echoed elsewhere (99).

The stating of such ambitions have sometimes gone hand in hand with recognition that doing so in practice would be frustrated by the demands of determining the risks associated with biological attacks. At times, these difficulties have been presented as surpassable through re-doubling efforts. For instance, in response to the recognition of uncertainty, the Royal Society’s *New Approaches to Biological Risk Assessment* advocated that “given the different nature of the risks across the spectrum and varying availability of data against which to derive or test mathematical models, a common approach should incorporate a range of specific assessments at points on the spectrum coupled with an overarching model to unify the resultant risk assessments” (100).

On other occasions, a more fraught relation between expectations and demands has been presented. In 2013, an international meeting of prominent government officials, practicing scientists, law enforcement officials, life science representatives, and others met at Wilton Park for a meeting titled “Dual-Use Biology: How to Balance Open Science with Security.” The outcome report of that meeting displays a desire, necessity, and possibility of definitive measures of risk and benefits as well as the challenges of producing them. With regard to the former, it was argued that:

Appropriate risk assessment should be part of the first phase of the research. Much work needs to be done to identify appropriate risk assessment factors relevant to DURC, taking into account the wide range of possible security concerns. In the future, a broader approach to risk could assess physical safety; economic security costs; diplomatic security; social and political stability; fear and anger and risk of research leading to the diminishing trust in government. It should also look at probability and take into account possible actors motives as well as intelligence on terrorist actors. Current DURC risk assessments have been largely “risk–benefit” analyses, and there is a need for much more comprehensive and quantitative risk assessments that specifically evaluate what could go wrong with certain research. The assessment should not be left solely to researchers and we need to incorporate all bodies and have a debate including governments which are responsible for crisis management and therefore need to consider responses (63).

And yet, while it was stated that “quantitative assessment sounds attractive because it feels evidence-based and hence more dependable and less open to counter-argument” (9), the Wilton Park report also noted that “the chances are that firm statistical data will be hard to come by, and that the sort of risks inherent in dual-use biological research cannot be quantified easily (which is not to say that they cannot be quantified at all)” (9). It was further contended that there is no “common understanding on how to conduct sound risk/benefit analysis; this is an issue between different states but also between different communities (scientific, security, etc.)” (7).

Though varying in their portrayal of the likelihood of achieving it, aspirations for comprehensive risk assessment methods have been made for years – this despite the lack of progress in that time toward specifying how risk–benefit analysis of research of concern could take place in practice. On this last point, the Wilton Park report contended that between “2005 and 2011 the NSABB established a risk/benefit methodology”; (3) a statement, which appears to conflate the process for the handling of risks and benefits with a methodology for determining risks and benefits.

Academic analysis of the prospects for risk–benefit assessment shares many of the same dynamics in treating research of concern as (more or less) susceptible to rational (often quantitative) analysis, but in practice being able to offer a limited articulation of how such assessment could be conducted (101, 102).

The need and prospect for elaborated formal risk–benefit assessment as a basis for decision making is not universally shared. Interviews undertaken by the author with one national biodefense establishment, for instance, indicated a preference for processes of dialog and professional judgment to identify concerns in contrast to the type of comprehension quantitative analysis sought elsewhere. The latter was judged as not necessary and not feasible.

Thus, the points above would suggest the continuing value placed with assessment processes has been promissory – the *future promise* of comprehensive assessments have been widely forwarded without explicit consideration of the ongoing inability to articulate how determinations of risk assessment could be made along the lines advocated (103). Such calls have shored up at least the prospect of the rational management of the dual-use concerns and thereby worked against arguments for rethinking the basic rationalistic framing of debates.

In contrast, this article has also offered reasons for questioning the prospect for achieving the types of comprehensive assessments envisioned. Arguably the situation is not simply one of uncertainty about the details of certain parameters associated with the type and extent of misuse risk nor is it the case that is only difficult to describe the likely outcomes of the malign application of research. Rather in many cases, both probabilities and outcomes are characterized by many unknowns and subject to different interpretations in such a way as to confound the devising of methods of assessment. If this appraisal is correct then it is necessary to foster other ways of understanding in order not to prematurely close down thinking. It is also necessary not to lend a false confidence to what is being grasped by existing review processes. For instance, the listing of funder and publisher review procedures has been forwarded at times as grounds for assurance about the level of scrutiny today (104). Whether that implication is warranted seems open to question given the argument above that the details about how assessments are being made makes it highly unlikely that expected risks would ever outweigh anticipated benefits.

## Alternative Possibilities

In recent decades, considerable effort has gone into asking how risks associated with science and technology can be handled more generally. A recurring theme from such investigation has been the need to recognize the fact that risk–benefit assessments are often of limited applicability in making decisions. When the outcomes and probabilities can be straightforwardly and consensually characterized, such methods can play a significant role in risk management. In the absence of such conditions though, reducing decision making to conventional risk–benefit analysis should not be seen as rational or reassuring (105).

In relation to the specific topic of this article, how then might we move away from the narrow question of whether this or that particular instance of science will likely result in more risks than benefits? One manner in which this has been done is by asking about the place of “precaution” in making sense of issues. The remainder of this paper considers what space can be opened up through taking inspiration from this topic.

While diverse in their formulations (see below), efforts to inject precaution into science and technology policy have usually shared the premise that definitive evidence of negative consequences need not be demonstrated to justify deliberation or even action (106). Instead, attempts have been made to ask what uncertainties, unknowns, and ignorances imply for who has to prove what to whom and for what purpose.

Precaution has become an overarching principle in national and international regulations such as the Cartagena Protocol on Biosafety, the Rio Conference on Environment and Development, and the Montreal Protocol on Substances that Deplete the Ozone Layer. And yet, despite the widespread reference to “the precautionary principle,” especially in environmental policy, the practical relevance of these types of orientations is disputed (107, 108).

Within biosecurity life science discussions, precautionary orientations to risk have been dismissed at times. As argued, for instance:

Using an alternative method such as the precautionary approach to try to overcome these problems would be quite inappropriate for governing dual use technologies. Although the precautionary approach casts a wide net, precautionary regulations over every potential technology that could be misused would be not only prove to be infeasible in the case of dual use research and technologies but may have a dramatic social costs through stigmatizing the legitimate applications of these technologies (109).

Despite what is implied in such an evaluation, precautionary ways of orientating to risk are diverse. Peterson spoke of this diversity in considering how these approaches differed in their answers to the questions:

- What level (threshold) of threat or potential for harm is sufficient to trigger application of the principle?
- Are the potential threats balanced against other considerations, such as costs or non-economic factors, in deciding what precautionary measures to implement?
- Does the principle impose a positive obligation to act or simply permit action?
- Where does the burden of proof rest to show the existence or absence of risk of harm?
- Is liability for environmental harm assigned and, if so, who bears liability? (110)

As implied by these questions, formulations of precaution still depend on the identification of risk, but they need not invest risk–benefit assessments with the definitiveness that is implied in dual-use discussions today.

Other attempts to map the range of precautionary orientations have set out taxonomies (111, 112). Luján and Todt, for instance, distinguish versions of precautionary principles according to how they handle scientific uncertainty about consequences, make judgments in relation to disputed harmful consequences, and view the controllability of technology (113). With these criteria, Luján and Todt offer three different interpretations.

* Under the “Risk-based Interpretation” the need for precaution enters when there is a credible basis for significant negative consequences, but a lack of scientific certainty about whether they will likely result. As such, precaution is a supplement to attempts to regulate through traditional forms of risk management.
* In the “Epistemological Limits Interpretation,” much more scope is given to the possibility of uncertainty or ignorance. Rather than ideally being able to be eliminated, they are treated as often prevalent and irresolvable. As such, decision making needs to make use of, but also go beyond, traditional risk assessment. That might entail, for example, not simply attempting to assess risks on a case-by-case basis, but instead adopting categorical orientations to classes of science and technology. Within the Epistemological Limits Interpretation, it is essential to learn as much as possible about (i) the presumptions guiding interpretations of risk where there is uncertainty and ignorance in order to make them a topic of consideration and (ii) the limits of science in order to ask if non-traditional methodologies might offer useful ways of handling risks. Through such actions, expectations about who has to prove what and to what standard might need to change.
* Finally, as part the “Technology Selection Interpretation,” precaution stands opposed to traditional forms of risk assessment. Typically within such orientations, categorical evaluations about the benefits and dangers of certain technologies are made (e.g., GM crops), and then the promotion or prohibition of whole trajectories of activities based on their risks or, even, lack of data about risks. Such sweeping decisions can be taken either to avoid the possibility of negative consequences or to promote positive social goals (such as sustainability).

Against this taxonomy, it is possible to suggest how dual-use discussions to date are already (albeit mainly implicitly) infused with precautionary-type reasoning. For instance, as argued previously, discussions about how to assess research of concern often start with presumptions – such as that dual-use risks need to be substantiated, whereas, in general benefits from research can be assumed – that shape assessments of what needs doing.

Alternative starting presumptions have been voiced elsewhere. In relation to the H5N1 controversy, two new former members of NSABB, Michael Osterholm and David Relman, contended that the risks at stake were so grave (catastrophic human pandemic) and benefits unclear, that “the precautionary principle” should be evoked to err on the side of not doing harm – meaning that the work led by Fouchier needed to be censored (114).

Another precautionary paralleled facet of responses has been the opting of categorical approaches requiring specific logics of decision making rather than case-by-case assessments. *A Framework for Guiding U.S. Department of Health and Human Services Funding Decisions about Research Proposals with the Potential for Generating Highly Pathogenic Avian Influenza H5N1 Viruses that are Transmissible among Mammals by Respiratory Droplets*, for instance, stipulates that there is a category of research that is different from others (115). Within the US Department of Health and Human Services, funding proposals that fall into this category must undergo review scrutiny wherein the work must meet certain criteria (such as that there is no feasible alternative method to address the same scientific question in a manner that poses less risk and that the information generated is anticipated to be broadly shared in order to advance global health).

### From decisions to processes

Up until this point in this article, precaution largely has been conceived as a factor in decision making. Precaution as a decision rule that prescribes action, however, is only one (and perhaps a highly) limited conceptualization of the notion. In practice, precautionary orientations to risk enacted to date have rarely provided definitive operational rules for making decisions or even stipulated clear cut criteria. Instead of being a rule for decision making, precaution can be thought about for what is implied for the *process* of deliberating risks. Consider a number of dimensions to this.

#### Examining foundations

With the acknowledgment given to uncertainties, ambiguities, and ignorances, attention should be directed at the starting points that shape understanding. These should be made explicit and a topic for reflection. In other words, the values underpinning interpretations to risks must be acknowledged and scrutinized. These may, for instance, have significant implications for how the burden of proof is distributed (105). In this sense, making scope for precaution itself does not imply that specific concerns take priority (for instance, preserving scientific development, environmental sustainability, avoiding a catastrophic pandemic, etc.), merely that the (likely varied and multiple) commitments for making sense of uncertainties, ambiguities, and ignorances be the subject of examination (116).

#### Shifting discussion terms

In fostering certain kinds of deliberation, precautionary-inspired deliberations can lend credibility and legitimacy to some arguments. In relation to how references to the precautionary principle entered into deliberations about conservation in fishing, for instance, it has been argued that the effects have been significant:

first by enhancing the credibility of certain types of arguments and diminishing that of others; second, by providing a framework within which conservationist arguments can be presented; and third, by pointing to interests and values other than those of states as legitimate objectives which the conservation regime should pursue (117).

Elsewhere precaution has diminished the credibility of narrow, notionally “scientific” forms of determining risks (108).

The need to reconsider the relevancy expertise in the process of making sense of the malign applications of science was given in an examination by Vogel of how US intelligence analysts assessed the H5N1 experiments (118). Her conclusions were three-fold:

First, U.S. intelligence analysts do not have adequate social and material resources to identify and evaluate the tacit knowledge, or know-how, that underpins dual-use experiments such as those in the H5N1 case. Second, they lack dedicated structures and methods to sort through the politics that characterize the use of technical expertise in such controversial biosecurity issues. Third, they require new types, structures, and assessments of expert knowledge to enable them to make more informed and balanced judgments of biosecurity threats (48, 80).

As part of enacting these recommendations, she contended that intelligence analysts need to be able to draw on a wider range of experts, including those in the social sciences.

#### Promotion of alternative methodologies

In maintaining the applicability of traditional forms of risk assessment are limited due to uncertainties, ambiguities, and ignorances, those adopting precaution orientations have sought alternative methods for making sense of risk. These have been either replaced or complement conventional assessments (105). Examples include scenario analysis, interval analysis, Q-method, horizon scanning, and societal impact assessment (119). Whereas conventional risks assessment might be done with the aim of weighing risks and benefits so as to make decisions, methods based on the recognition of incertitude aim to understand the limits of what is known, aid professional judgments, identify starting assumptions, reframe debates, and promote dialog and interaction. Making use of such methods can result in the participation of a different range of individuals than conventional risk assessment. Along these lines, as part of the analysis of H5N1, Vogel suggested how intelligence analysis could benefit from new forms of engagement that tested its limitations (118).

One area where these dimensions of precaution come together is public engagement. Within precautionary orientations, the overall attention to the limits of scientific certitude in determining risks and their acceptability opens a space for a wide range of contributions; including by those in publics. As argued, though:

“broadening out” of the social appraisal of technology that precaution may also be seen to entail a more generally *comprehensive* approach to decision making. A key consideration here concerns the many ways in which precaution is inherently interlinked with participatory approaches. This is not only as an aspiration to enhanced democracy. Nor is it just about fostering greater public trust or education. Far from second-guessing technical expertise with irrational public anxieties, precautionary participation is a matter of improved analytical rigor (emphasis in original) (105)

It would be difficult to over-estimate how much of the dual-use discussion to date has cast the public as a threat to science due to the potential for “misunderstanding” and sensationalism. As detailed elsewhere, within the Communication Working Group of NSABB, “the public” has come to occupy a central (if not the most prominent) place due to fears of public misunderstanding and sensationalism (120). In response to fears about the public, advisory documents such as NSABB”s *Proposed Framework for the Oversight of Dual-Use Life Sciences Research: Strategies for Minimizing the Potential Misuse of Research Information* provide many points about the need to message the publication of dual-use research so as to highlight the safeguards on research and its benefits.

Elsewhere in science policy over the last two decades, attempts have been made to recast the public away from being a problem for the acceptance of science and technology. Instead, efforts have been made to promote the engagement of the varied and numerous publics within a dialog (121). Public participation has been sought, for instance, as a means to highlight the importance of social values, to challenge technocratic framings, to identify alternative paths for the development of technology, and to promote what is coined as “responsible innovation” (122). While realizing such aspirations in practice is highly demanding, a more positive and arguably more productive role for the public is envisioned within them that typifies dual-use discussions to date (123).

## Conclusion

This article has examined the origins, emergence, resurrection, and implications of the category “research of concern.” Throughout, attention has been given to a curiosity: the rarity that anything is identified as “of concern.” The previous argument would suggest that the outcomes of review procedures enacted to date are the result of contingent practices that are consequential in the manner they structure a sense of what is going on and why, as well as what needs doing and by whom. In theory, this situation leaves open the possibility that a different pattern of review outcomes might take place if alternative conditions are in place.

More critically, as part of making the case for contingency, the preceding argument has questioned the continuing prominence given to conventional rationalistic “risk–benefit” assessment in managing the dual-use dimension of the life sciences. The notion of “weighing risks and benefits” may have substantial symbolic purchase for some, but arguably has limitations as a way of framing responses to research of concern. Without an acknowledgment of these, it is possible that a misplaced confidence is invested in reviews as currently conceived and that alternative policy possibilities are not sought out. Like other complex social and scientific issues, arguably it would not be wholly unfair with respect to the topic of this article to contend that “not only is the solution unknown, but the problem itself is initially not well defined, and the values that ought to drive its investigation and the valid methods to do so are unknown, unclear, or in dispute, as are the set of applicable theoretical models, the solution set, and the criteria for successful resolution” (124).

In reply, this article has outlined one set of different possibilities associated with “precaution.” Though varied in their formulations, precautionary orientations generally begin with the aim of acknowledging conditions of uncertainty, ignorance, and ambiguity in order to ask how issues can be sensibly approached nevertheless. As argued, adopting such a starting basis could open spaces for alternative ways of thinking and responding to a set of issues that are bound to uncomfortably accompany the life sciences into the future.

## Conflict of Interest Statement

The author declares that the research was conducted in the absence of any commercial or financial relationships that could be construed as a potential conflict of interest.
